# Editorial Note: Where octagonal geometry meets chaos: A new S-Box for advanced cryptographic systems

**DOI:** 10.1371/journal.pone.0354208

**Published:** 2026-07-21

**Authors:** The PLOS One Editors

The *PLOS One* Editors issue this Editorial Note to inform readers that the article cited as Reference 34 was retracted before the publication of this article [1].

Additionally, PLOS has concerns about the article’s authorship and the peer review process. Readers are encouraged to approach the article [1] with careful consideration.

The Editors do not have concerns regarding the authors’ conduct during the peer review process.
